# Role and Mechanisms of Mitophagy in Liver Diseases

**DOI:** 10.3390/cells9040837

**Published:** 2020-03-31

**Authors:** Xiaowen Ma, Tara McKeen, Jianhua Zhang, Wen-Xing Ding

**Affiliations:** 1 Department of Pharmacology, Toxicology and Therapeutics, University of Kansas Medical Center, 3901 Rainbow Blvd., Kansas City, KS 66160, USA; xma3@kumc.edu (X.M.); tmckeen@kumc.edu (T.M.); 2 Department of Pathology, Division of Molecular Cellular Pathology, University of Alabama at Birmingham, 901 19th street South, Birmingham, AL 35294, USA; jianhuazhang@uabmc.edu

**Keywords:** alcohol, autophagy, mitochondria, NAFLD, Parkin, Pink1

## Abstract

The mitochondrion is an organelle that plays a vital role in the regulation of hepatic cellular redox, lipid metabolism, and cell death. Mitochondrial dysfunction is associated with both acute and chronic liver diseases with emerging evidence indicating that mitophagy, a selective form of autophagy for damaged/excessive mitochondria, plays a key role in the liver’s physiology and pathophysiology. This review will focus on mitochondrial dynamics, mitophagy regulation, and their roles in various liver diseases (alcoholic liver disease, non-alcoholic fatty liver disease, drug-induced liver injury, hepatic ischemia-reperfusion injury, viral hepatitis, and cancer) with the hope that a better understanding of the molecular events and signaling pathways in mitophagy regulation will help identify promising targets for the future treatment of liver diseases.

## 1. Introduction

Autophagy (or macroautophagy) involves the formation of a double membrane structure called an autophagosome. Autophagosomes bring the enveloped cargoes to the lysosomes to form autolysosomes where the lysosomal enzymes consequently degrade the cargos [1,2]. Autophagy is generally induced in response to the starvation conditions to generate biomolecules for cell survival purposes [1,2,3]. Autophagic degradation is split into two categories, selective or nonselective. Non-selective autophagy generally occurs upon nutrient deprivation, which causes the widespread breaking down of cytoplasmic components, supplying cells with nutrients for survival purposes. Selective autophagy targets the misfolded protein aggregates, damaged and excess organelles, endoplasmic reticulum (ER) and lipid droplets, and invading bacteria and viruses. Accumulating evidence indicates that selective autophagy plays a vital role in the regulation of liver homeostasis. It is therefore not surprising that impaired selective autophagy, specifically mitophagy (selective autophagy for damaged/excess mitochondria), has recently been shown to contribute to alcoholic liver disease (ALD) and non-alcoholic fatty liver disease (NAFLD), drug-induced liver injury, hepatic ischemia-reperfusion injury (IR), viral hepatitis, and liver cancer [4,5,6,7,8]. The role of autophagy and mitochondrial dysfunction in liver diseases has been excellently summarized in previous review papers [4,5,8,9,10], this review will focus on the emerging roles and the regulatory mechanisms of mitophagy in the liver.

## 2. Selective Autophagy Receptors

Selective autophagy, including mitophagy, aids in the removal of damaged or excess organelles and protein aggregates, and requires specific autophagic receptors. Autophagy receptor proteins typically bind to cargo molecules and autophagy-related protein 8 (Atg8)/microtubule-associated protein 1A/1B light chain (LC3) simultaneously, acting as a bridge between the target cargo molecule and nascent autophagosome during the early stages of autophagy. These receptor proteins generally have the conserved Atg8 and LC3 interacting region (LIR) that consists of the core motif (W/F/Y-X-X-L/V/I). Currently, two types of autophagy receptors have been defined: soluble and membrane-anchored autophagy receptors. Soluble autophagy receptors (SARs) do not have a membrane translocation domain but have the ubiquitin-binding domain. The most studied SARs are sequestosome 1 (SQSTM1)/p62 (hereafter referred to as p62), the neighbor of the BRCA1 gene 1 (NBR1), nuclear domain 10 protein 52 kDa (NDP52), optineurin and TAX1 binding protein 1 (TAX1BP1). These SARs generally have one to two LIR motifs and one ubiquitin-binding domain at the C-terminus. During selective autophagy, the selective cargos, including proteins, organelles, and invading bacteria are often ubiquitinated. The SARs bind to their cargo molecules via the ubiquitin-binding domain and attach themselves together with these cargo molecules to the autophagosome membrane through LIR-mediated interaction with lipidated LC3. The membrane-anchored autophagy receptors (MARs) already reside on the membrane organelles and do not directly bind with ubiquitin. For instance, Bcl2/adenovirus E1B 19 kDa protein-interacting protein 3 (BNIP3), Nix/BNIP3L, FUN14 domain containing 1 (fundc1), Bcl2-like 13 (Bcl2L13) and prohibitin 2 (PHB2), are all mitochondrial membrane proteins that directly interact with LC3 by their LIR motifs that recruit the LC3 positive phagophore to the mitochondria in order to initiate mitophagy [11,12,13,14]. In addition to the mitochondria autophagy receptors, there are several endoplasmic reticulum-anchored autophagy receptors including family with sequence similarity 134, member B (FAM134B), SEC62, reticulon 3 (RTN3), cell cycle progression 1 (CCPG1), atlastin GTPase 3 (ATL3) and testis expressed 264 (TEX264). These receptors have been noted to also act as specific ER-phagy receptors by directly interacting with LC3 [15,16,17]. More autophagy receptor-related information can be found in these recent excellent review papers [18,19,20].

## 3. Mitophagy Signaling Pathways

### 3.1. PINK1-Parkin-Dependent Mitophagy

The term “mitophagy” refers to selective autophagy for mitochondria, first coined by John Lemasters, who observed depolarized mitochondria entering into acidic lysosomal compartments in hepatocytes [21,22]. In 2008, Richard Youle’s group from NIH reported that Parkin, an E3 ubiquitin ligase encoded by the gene Park2, translocates to depolarized mitochondria by promoting its engulfment by autophagosomes resulting in the selective removal of these damaged mitochondria by mitophagy [23]. Subsequently, Parkin was found to be recruited to damaged mitochondria by phosphatase and tensin homolog-induced putative kinase 1 (PINK1) to initiate mitophagy [24,25,26]. Since then, the PINK1-Parkin-mediated mitophagy pathway has been widely studied and knowledge regarding this pathway has been markedly enriched. PINK1 is generally undetectable in healthy mitochondria because it is cleaved by presenilin associated, rhomboid-like (PARL) protein after it is imported into the mitochondrial matrix [27]. The cleaved PINK1 fragments are then released into the cytoplasm where they are degraded by the ubiquitin proteasome system through the N-end rule pathway and the cleaved cytosolic PINK1 fragments inhibit Parkin translocation to mitochondria by directly interacting with Parkin [28,29]. However, in damaged depolarized mitochondria, PINK1 is stabilized on the outer mitochondrial membraneand phosphorylates both ubiquitin and Parkin at Ser65 in order to activate Parkin’s E3 ligase activity, and recruits Parkin from the cytosol to mitochondria [27,30,31,32]. Once on the mitochondria, Parkin increases the ubiquitination of the outer mitochondrial membrane proteins that bind with SARs to further recruit autophagosomes to damaged mitochondria. This information indicates that there are two cellular pools of PINK1, which may differentially regulate Parkin translocation and mitophagy. Cytosolic PINK1 fragments inhibit Parkin translocation whereas mitochondrial PINK1 promotes Parkin translocation and mitophagy. The most commonly studied Parkin substrates in the outer mitochondrial membrane are the mitochondrial fusion proteins mitofusin 1 (Mfn1) and mitofusin 2 (Mfn2), the mitochondrial trafficking protein Miro1, translocase of outer mitochondrial membrane 20 (TOM20), and voltage-dependent anion channel (VDAC) [33]. Once ubiquitinated, Mfn1 and Mfn2 are degraded by the proteasome resulting in mitochondrial fragmentation [34,35,36,37]. Mitochondrial fragmentation not only facilitates the segregation of damaged mitochondria from healthy mitochondria but also enables mitophagy initiation because smaller mitochondria are more easily engulfed by autophagosomes [34,38,39,40,41]. Moreover, PINK1/Parkin-mediated ubiquitination of VDAC recruits the SARs, such as p62 to the mitochondria. Next, p62 is further recruited to LC3 positive autophagosomes to initiate mitophagy, although it should be noted that the role of p62 in mitophagy is still controversial and may be cell type dependent [36,42,43,44]. Parkin also ubiquitinates BCL2/adenovirus E1B 19 kDa interacting protein 3-like (BNIP3L, also known as NIX), which allows NIX to recruit another SAR protein, the neighbor of BRCA Gene 1 (NBR1), to the damaged mitochondria to help shuttle them to the autophagosome for degradation [45]. In addition to the mitochondrial outer membrane proteins, prohibitin 2 (PHB2), a mitochondrial inner membrane protein, also plays a role in mitophagy by binding to LC3 through the LIR domain upon mitochondrial depolarization and proteasome-dependent outer membrane rupture in a Parkin-dependent manner [46]. Parkin also interacts with autophagy/beclin-1 regulator-1 (Ambra1) upon mitochondrial depolarization, and Ambra1 then further activates class III PI3K for autophagosome formation surrounding these mitochondrial clusters for their clearance [47]. Notably, mitochondria can actively move via the motor/adaptor complex, which consists of Miro (RhoT1/2) and milton (also called TRAK1/2) located on the mitochondrial surface. PINK1 phosphorylates Miro resulting in the proteasomal degradation of Miro in a Parkin-dependent manner. Following the removal of Miro from mitochondria, kinesin detaches from the mitochondrial surface that leads to the stall and arrest of the mitochondria, which facilitates the engulfment of the mitochondria by autophagosomes [48]. Mitophagy is also able to be negatively regulated by two mitochondrial localized deubiquitin enzymes, ubiquitin-specific peptidase 30 (USP30) and ubiquitin-specific peptidase 15 (USP15). These enzymes remove the Parkin-mediated ubiquitin from the damaged mitochondria [49,50].

### 3.2. Parkin-Independent Mitophagy

Accumulating evidence indicates that mitochondria can be removed by autophagy independent of Parkin. Several autophagy receptor proteins are also mitochondrial resident proteins, such as BNIP3, NIX, and FUNDC1, and are induced under hypoxic conditions. These receptor proteins further recruit autophagosomes to mitochondria through direct interaction with LC3. The BH3-only protein BNIP3 has dual functions in regulating cell death and mitophagy. Phosphorylation of Ser17 and Ser24 on BNIP3 LIR motif positively regulates its binding to LC3, promoting pro-survival mitophagy instead of apoptosis in mammalian cells [51,52]. NIX-mediated mitochondrial elimination was identified during erythrocyte maturation, which also can be activated under in vitro hypoxic conditions [53,54,55]. Bcl2-L-13, a yeast Atg32 homolog, is a single transmembrane domain outer mitochondrial membrane protein that belongs to the Bcl-2 family protein. Bcl2-L-13 has two WXXI motifs, allowing it to bind to LC3 and promotes mitophagy in HEK293 cells after carbonyl cyanide m-chlorophenyl hydrazine (CCCP) treatment independent of Parkin [56]. FUNDC1 is phosphorylated on Tyr18 by the Src kinase and dephosphorylated on Ser13 by the mitochondrial phosphatase phosphoglycerate mutase family member 5 (PGAM5) [57,58]. Dephosphorylated FUNDC1 has a higher binding affinity with LC3 to facilitate mitophagy in response to hypoxia [57]. In addition, hypoxia increases Unc-51 like autophagy activating kinase 1 (ULK1) expression and translocation to fragmented mitochondria where it directly interacts with FUNDC1 to be phosphorylated at Ser17. Interestingly, phosphorylated FUNDC1 at Ser17 also enhances its binding with LC3 to promote mitophagy [59]. Therefore, it seems that phosphorylation or dephosphorylation of FUNDC1 at different sites can both enhance its binding with LC3 to promote mitophagy. Furthermore, E3 ligase membrane-associated ring-CH-type finger 5 (MARCH5) causes FUNDC1 ubiquitination and subsequent proteasomal degradation, protecting mitochondria from autophagic destruction [60].

In addition to the protein autophagy receptors, lipids can directly bind LC3 and act as autophagy receptors. For instance, cardiolipin (CL), a mitochondrial specific phospholipid, which is predominantly located on the inner mitochondrial membrane but can translocate to the outer mitochondrial membrane in response to various mitochondrial stresses such as the exposure to rotenone, staurosporine, and 6-hydroxydopamine [61]. The outer mitochondrial membrane and CL directly interact with LC3 to trigger mitophagy [61,62]. In addition to CL, ceramide, a bioactive sphingolipid, directly binds LC3 to recruit autophagosomes to the mitochondria resulting in cell death, likely due to the induction of lethal mitophagy in cancer cells [63].

Notably, several other E3 ligases have been reported to be involved in mitophagy in addition to Parkin. In Drosophila and mammalian cells, mitophagy is induced in depolarized mitochondria by E3 ligase Mul1 acting parallel to Parkin to ubiquitinate the outer mitochondrial membrane Mfn1/Mfn2 proteins. In one study, single mutant PINK1, Parkin or Mul1 flies were compared to double-mutant flies (PINK1/Mul1 and Parkin/Mul1), with the double-mutant flies demonstrating worsened phenotypes (i.e., increased mortality, muscle degeneration, damaged mitochondria, and reduced levels of ATP). Knockdown or overexpression of Mul1 in Parkin-expressing HeLa cells did not affect Parkin translocation to the mitochondria following mitochondrial depolarization [64], suggesting that Mul1 may act independently of Parkin.

**Figure 1:**
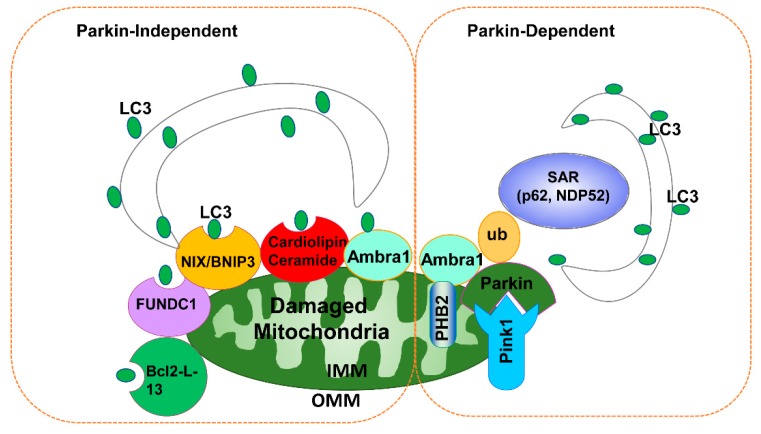
Parkin-dependent and Parkin-independent mitophagy. In the presence of Parkin, damaged/depolarized mitochondria (e.g., following CCCP treatment) stabilize PINK1 that recruits Parkin to the mitochondria. Once on the mitochondria, Parkin promotes ubiquitination of outer mitochondrial membrane proteins, which serve as binding partners for SAR, such as p62 or NDP52. SAR acts as an adaptor molecule through direct interaction with LC3 to recruit autophagosomal membranes to the mitochondria. The mitochondrial inner membrane protein PHB2 also binds to LC3 through its LIR domain upon mitochondrial depolarization after proteasome-dependent outer mitochondrial membrane rupture in a Parkin-dependent manner. For the Parkin-independent pathway, damaged mitochondria (particularly under hypoxia conditions) increase the expression of FUNDC1, NIX, and BNIP3, which may in turn recruit autophagosomes to mitochondria by direct interaction with LC3 through their LIR domains. Upon mitochondrial depolarization, Bcl-2-L13 also promotes mitophagy independent parkin. Upon toxin or drug-induced mitochondrial damage, mitochondrial lipid Cardiolipin and ceramide also bind to LC3 and promote mitophagy independent of Parkin. Notably, Ambra1 may promote mitophagy in both Parkin-dependent and Parkin-independent manners. IMM, inner mitochondrial membrane; OMM, outer mitochondrial membrane; SAR, soluble autophagy receptor; ub, ubiquitin.

Therefore, in the absence of Parkin, Mul1 may be an important compensatory pathway for mitophagy. Smurf1 is an E3 ubiquitin ligase that may also play a role in mitophagy. The C2 domain of Smurf1 is required for engulfment of damaged mitochondria by autophagosomes and it was found that Smurf1-deficient mice have an increased accumulation of damaged mitochondria in the heart, brain, and liver [65]. The E3 ubiquitin ligase glycoprotein 78 (Gp78) plays a role in endoplasmic reticulum-associated degradation. Overexpression of Gp78 increases the ubiquitination of Mfn1/Mfn2 leading to their degradation via proteasomes resulting in increased mitochondrial fragmentation and mitophagy. In contrast, knockdown of Gp78 increases mitofusin levels and reduces depolarization-induced mitophagy independent of Parkin [66]. The E3 ubiquitin ligase HUWE1 promotes mitophagy by enhancing IKKα-mediated AMBRA1 phosphorylation and Mfn2 degradation. Phosphorylated AMBRA1 at Ser1014 enhances its binding with LC3 and in turn, promotes mitophagy independent of PINK1/Parkin [67]. Therefore, Ambra1 may be involved in mitophagy in both Parkin-dependent and Parkin-independent pathways. In addition, PINK1 was reported to recruit mitophagy receptors and activate mitophagy directly, independent of Parkin in Hela cells. Activated PINK1 accumulates on damaged mitochondria and phosphorylates ubiquitin on its substrates, which further recruit two SARs, OPTN and NDP52. Autophagy initiation factors, such as ULK1, double FYVE-containing protein 1 (DFCP1) and WD repeat domain phosphoinositide-interacting protein 1 (WIPI1) are subsequently recruited to mitochondria by these receptors to initiate PINK1-dependent mitophagy [68]. Taken together, it is clear that mitophagy can be induced by either Parkin-dependent or Parkin-independent pathways (Figure 1). Readers who are interested in this topic are encouraged to read more other excellent recent review articles [69,70,71].

### 3.3. Other Mitochondrial Quality Control Mechanisms

In addition to the mitophagy pathway, mitochondria have their own proteases that recognize misfolded or oxidized mitochondrial proteins resulting in mitochondrial degradation. Similar to ER stress response, mitochondria have their own stress response called the mitochondrial unfolded protein response (UPR^mt^) [72]. The dramatically increased mitochondrial protein synthesis and import rates often lead to the accumulation of misfolded and unassembled proteins beyond the folding capacity of mitochondria. This triggers a retrograde signaling pathway from the mitochondria to the nucleus resulting in the upregulation of mitochondrial chaperones and proteases in order to gain mitochondrial homeostasis. Activating transcription factor 5 (ATF5), is a transcription factor that localizes in both mitochondria and nuclei. In response to mitochondrial dysfunction, ATF5 translocates to the nuclei and induces the expression of several mitochondrial chaperones including heat shock protein 70 (HSP70), HSP60, HSP10 and mitochondrial protease Lon protease homolog 1 (LONP1), resulting in the re-establishment of mitochondrial homeostasis [72,73]. If the stress exceeds the capacity of UPR^mt^, other mechanisms such as mitophagy will be induced to remove the damaged mitochondria.

Oxidized mitochondrial proteins can be enwrapped in small vesicle buds off of the damaged mitochondria under conditions of mitochondrial oxidative stress, this is called mitochondria-derived vesicles (MDVs) [74]. The formation of MDVs is induced by mitochondrial reactive oxygen species (ROS) but not mitochondrial depolarization. Notably, formation of MDVs is also dependent on the PINK1 and Parkin proteins. The MDVs carry the oxidized mitochondrial proteins to the lysosome where fusion occurs resulting in the degradation of mitochondrial proteins. The MDVs may represent an alternative faster mitochondrial quality control mechanism than canonical mitophagy that requires the biogenesis of autophagosomes. MDVs selectively remove the damaged proteins without removing the whole mitochondrion and thus can help to preserve the mitochondrial contents and functions [74,75]. More recently, it is reported that MDVs carry the mitochondrial-derived H_2_O_2_ and fuse with phagosomes that are enwrapping invading bacteria resulting in the demise of bacteria upon infection [76].

**Figure 2:**
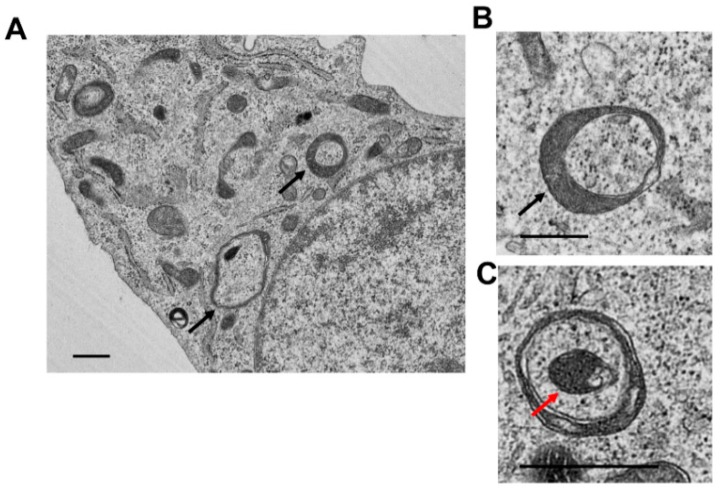
The structure of the mitochondrial spheroids under electron microscopy (EM). Wild-type mouse embryonic fibroblasts (MEF) were treated with CCCP (20 µM) for 16 h. Cells were fixed and further processed for EM analysis. Representative EM images of a MEF (**A**), typical mitochondrial spheroid structures (**B**,**C**). Black arrows denote mitochondrial spheroids and the red arrow denotes mitochondria spheroid-enwrapped mitochondria.

In addition, formation of mitochondrial spheroids may be another type of alternative quality control mechanism [77,78,79]. Mitochondrial spheroids have a ring or cup-shaped morphology with a squeezed mitochondrial matrix. Interestingly, mitochondrial spheroids also enwrap intracellular organelles such as endoplasmic reticulum, lipid droplets, or even other mitochondria as well as cytosolic proteins (Figure 2). Mitochondrial spheroids have been observed in cultured primary cells, transformed cells and tumor cells as well as in mouse liver and neuron tissues [77,78,80,81,82]. Although mitochondrial spheroids have been shown to have also acquired some lysosome proteins but whether their contents are degraded within the mitochondrial spheroid lumen remains to be determined. In addition, whether the mitochondrial spheroids can reverse back to normal mitochondria, or the entire mitochondrial spheroid structures are removed by lysosomes is not known and needs to be studied in the future.

**Figure 3:**
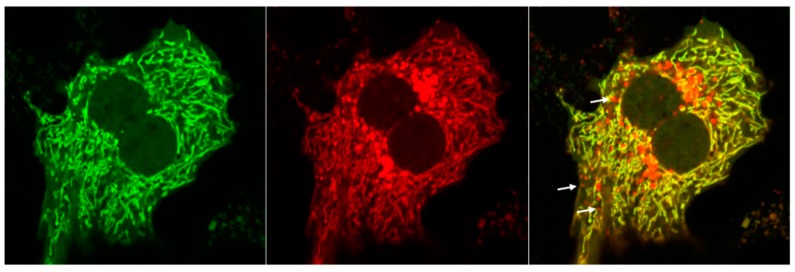
Monitoring mitophagy in primary hepatocytes using Cox8-GFP-mCherry. Primary mouse hepatocytes were infected with adenovirus-Cox8-GFP-mCherry (10 MOI) for 72 h followed by confocal microscopy. Arrows denote red-only autolysosome-enwrapped mitochondria.

Fission and fusion serve as two essential quality control mechanisms to favor the segregation and removal of damaged mitochondrial components in order to achieve homeostasis. Dysfunctional mitochondria may lose their fusion capacity by inactivating fusion or activating fission machinery to prevent the damaged components from incorporating into the healthy mitochondrial network [39]. The segregated damaged mitochondria are often depolarized, triggering subsequent mitophagy for their degradation. Mitochondrial fusion is mediated by several fusion proteins including Mfn1, Mfn2 and optic atrophy 1 (Opa1). Mfn1 and Mfn2 are mitochondrial outer membrane GTPases that mediate the fusion reaction between the outer membranes, while the inner membrane protein, Opa1, is mainly responsible for the fusion of mitochondrial inner membranes [83]. Mitochondrial fission in mammals is mediated by the cytosolic protein dynamin-related protein (Drp1), which can translocate to the outer mitochondrial membrane to constrict mitochondria leading to their eventual division and mitochondrial fragmentation. It is thought that these fragmented mitochondrial pieces are easier for the autophagosomes to envelope in order to proceed with removal. Several mitophagy receptor proteins interact with Drp1, Mfn1/2 or Opa1 as well to collaborate mitochondrial dynamics and mitophagy [56,84,85]. The depletion of Drp1 is associated with an increased mitochondrial size and impaired mitophagy, while the simultaneous loss of Drp1 and Opa1 reestablishes these events in mouse liver [86]. Indeed, Drp1 deficient mice were found to have decreased mitophagy in their cardiomyocytes resulting in lethal heart failure [87]. To confirm the notion that fragmented mitochondria are favorable for mitophagy, we used the tandem Cox8-GFP-mCherry as a readout to monitor mitophagy in primary cultured mouse hepatocytes. The principle of this assay is based on the fact that the green fluorescence of GFP generally quenches, whereas mCherry fluorescence is relatively stable at the cellular acidic compartments (such as autolysosomes that have low pH values) [88]. Therefore, normal mitochondria generally show a yellow color, whereas autolysosome-enwrapped mitochondria will only display red fluorescence. As can be seen, the red-only mitochondria were readily detected in cultured WT hepatocytes, which may reflect the basal levels of mitophagy. Interestingly, it appeared that all the red-only mitochondria are fragmented mitochondria and no elongated red-only mitochondria were ever observed using this assay in the hundreds of cells that we have assessed (Figure 3). These data generally support the current notion that smaller fragmented mitochondria are favorable for turnover via mitophagy. However, it should also be noted that Drp1 may not be essential for mitophagy as mitochondrial division and mitophagy can also occur in the absence of Drp1 [89,90], suggesting the possible existence of Drp1-independent mitochondrial fragmentation. Future work will be critical for the identification of these mitochondrial division machinery independent of Drp1. Nevertheless, it is reasonable to speculate that modulation of mitochondrial dynamics may be a potential therapeutic target for several liver diseases that are caused by damaged mitochondria such as drug-induced liver injury or alcohol-associated liver disease (as discussed below).

The current known mitophagy receptors in mammalian cell mitophagy is summarized in Table 1. It should be noted that most of the above mentioned mitophagy signaling pathways and receptors were discovered from in vitro cultured cells. In spite of limited in vivo mitophagy studies likely due to the technical challenges, a few studies of mitophagy has been reported in mouse heart and muscle [91,92]; kidney [93]; lung [94] and neurons [95]. Below we will focus on the discussion of mitophagy in the liver and liver diseases.

The current known mitophagy receptors in mammalian cell mitophagy is summarized in Table 1. It should be noted that most of the above-mentioned mitophagy signaling pathways and receptors were discovered from in vitro cultured cells. Despite limited in vivo mitophagy studies likely due to the technical challenges, a few studies of mitophagy have been reported in mouse heart and muscle [98,99]; kidney [100]; lung [101] and neurons [102]. Below we will focus on the discussion of mitophagy in the liver and liver diseases.

## 4. Mitophagy in Liver Diseases

### 4.1. Mitophagy in ALD

In the United States, 48% of all liver cirrhosis are due to ALD, which is also a major contributor to liver disease-related mortality in other countries [103]. Alcohol exposure can cause mitochondrial dysfunction in the liver, and chronic ethanol administration has been shown to inhibit the synthesis of mitochondrial respiratory complex proteins [104]. Ethanol lowers the capacity for oxidative phosphorylation, which conditions for ROS production. Mitochondrial DNA, which encodes 13 subunits of the electron transport chain (ETC) and ATP synthase, is vulnerable to elevated oxidative stress due to its proximity to the inner mitochondrial membrane, which is the major intracellular source of ROS [105,106]. It has been suggested that just one single dose of alcohol can cause mitochondrial DNA degradation in mice [107]. Rats fed with ethanol chronically showed enhanced 8-hydroxydeoxyguanosine (8-OHdG) accumulation and mitochondrial DNA (mtDNA) strand breaks in the liver [108]. Ethanol-induced damage to mtDNA, if not adequately repaired, will impair cellular energy metabolism and enhance ROS formation, leading to propagation of cell damage [109]. Strong evidence indicates that oxidative stress and mitochondria dysfunction are central to the pathogenesis of ALD [110,111]. It was observed in both acute and chronic ethanol consumption, that mitophagy serves as a protective mechanism in alcohol-induced liver injury by eliminating dysfunctional mitochondria [112,113]. The genetic depletion of Parkin accelerates acute and chronic and also, acute alcohol binge-induced liver injury and steatosis, which is likely due to impaired mitophagy in hepatocytes [114]. Compounds that are able to restore mitophagy inhibited by alcohol are able to attenuate liver injury in chronic alcohol feeding models [115,116]. These results indicate the protective role of mitophagy in alcoholic liver injury, and how targeting mitophagy may be a prospective therapeutic approach of ALD [117,118].

The effects of alcohol on mitophagy depend on the duration and amount of alcohol exposure. Increased mitophagy was reported in an acute alcohol binge rat model, which can be mediated by either the PINK1/Parkin or BNIP3 signaling pathways [119,120]. Hepatocellular mitochondria depolarize broadly to facilitate hepatic ethanol metabolism after immediate alcohol administration, which triggers a series of mitophagy regulating signals to promote mitophagy as a compensative response [121]. Therefore, elevated mitochondrial oxidative damage is concurrent with enhanced mitophagy in hepatocytes at this stage. Chronic alcohol feeding resulted in prominent dysfunctional mitochondria accumulation, which exceeds the processing ability of mitophagy. Decompensated mitophagy causes the release of mitochondrial damage-associated molecular patterns (mtDAMPs) that promote inflammatory and profibrogenic response, which in turn impair hepatic mitophagy, contributing to the pathological progression of ALD [121]. Impaired mitophagy was reported in the majority of chronic alcohol feeding cases [115,116,122], underscoring its potential critical role in the pathogenesis of ALD.

**Figure 4:**
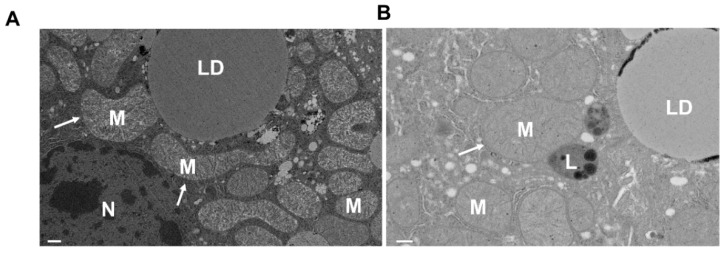
Megamitochondria in mouse livers after chronic plus binge alcohol feeding. Two three-month-old C57BL/6 mice were subjected to the chronic-plus-binge (Gao-binge) alcohol model. Liver sections were fixed and electron microscopy followed (**A** and **B** from Gao-binge alcohol-fed mouse livers). Arrows denote megamitochondria. L, lysosome; LD, lipid droplet; M, mitochondria; N, nucleus. Bar: 50 nm.

Interestingly, mitochondrial fission protein Drp1 was activated in primary mouse hepatocytes treated with ethanol for 48 h, leading to mitochondrial fragmentation, which is associated with depressed FUNDC1-mediated mitophagy [122]. However, the causal role of Drp1 in the changes of hepatic mitophagy after alcohol has not been examined in this study [122]. In contrast, increased megamitochondria formation has been identified in patients with ALD and chronic alcohol feeding animal models [123,124,125]. We also found an increased number of hepatic megamitochondria in mouse livers after chronic-plus-binge alcohol (so-called Gao-binge) feeding (Figure 4) [126]. Therefore, the exact role of Drp1 and mitochondrial fission in regulating mitophagy and hepatic energy metabolism in ALD remains to be further explored in the future.

### 4.2. Mitophagy in NAFLD

NAFLD is a hepatic manifestation of the metabolic syndrome consisting of a spectrum of liver disorders that begins with simple steatosis, which can progress to nonalcoholic steatohepatitis (NASH), fibrosis, cirrhosis, and hepatocellular carcinoma (HCC) at a later stage [127]. The pathogenesis of NAFLD arises from aberrant lipid metabolism in the liver, which is characterized by increased lipogenesis and elevated hepatocytes free fatty acid (FFA) uptake caused by insulin resistance. The lipotoxicity triggers a series of second hits including mitochondrial dysfunction, excessive oxidative stress, ER stress, inflammation, and profibrogenic response, predisposing the liver to high-risk conditions [128]. Considering the key role of the mitochondria in fatty acid metabolism and energy generation, impaired mitochondrial function is thought to be a hallmark of NAFLD [129]. Enlarged and swollen hepatocellular mitochondria with a loss of cristae were first described in patients with NASH, and these anomalous mitochondria were subsequently demonstrated to have decreased activity of respiratory chain enzyme complexes [130,131,132].

Resembling ALD, the removal of damaged mitochondria through mitophagy is widely regarded as a protective mechanism in long-term NAFLD development. Defective mitophagy was reported in both high-fat diet (HFD)-induced in vivo mouse models and in vitro cultured cells treated with oleic acid (OA) or palmitic acid (PA), associating with a series of NAFLD-related phenotypes including increased fat accumulation, elevated oxidative stress, and inflammation [133,134,135,136,137,138,139,140]. Several signaling pathways were involved in the regulation of mitophagy in NAFLD, and if manipulated properly could potentially restore mitophagy in hepatocytes and eventually improve metabolic outcomes. The expression of Acyl-CoA:lysocardiolipin acyltransferase-1 (ALCAT1) was up-regulated in an 18-week HFD-induced NAFLD mouse model. Genetically removing ALCAT1 can restore mitophagy in isolated hepatocytes, improving mitochondrial architecture and mtDNA fidelity, and preventing the onset of NAFLD in mice [133]. The mechanisms by which ALCAT1 negatively regulates mitophagy in NAFLD still remains elusive and needs further studies. Genetic ablation of macrophage stimulating 1 (Mst1), a novel cell survival regulator, has been shown to attenuate HFD-induced liver injury and sustain hepatocyte viability, which is likely due to the stimulation of PINK1/Parkin-mediated mitophagy [134]. Consistently, pharmacologically enhancing PINK1/Parkin-dependent mitophagy by the plant flavonol quercetin was reported to alleviate HFD-induced hepatic disorders in a 10-week feeding mouse model [137]. However, studies from mice with genetic deletion of Parkin yield very surprising results. Kim et al. reported that Parkin knockout mice were protected from 6-week HFD-induced obesity, hepatic steatosis, and insulin resistance [141]. Further mechanical studies implied that Parkin conferred these effects via ubiquitin-mediated stabilization of the lipid transporter CD36 in the liver [141,142]. Interestingly, in an acute HFD feeding model, impaired intestinal lipid absorption was found in Parkin knockout mice as evidenced by increased fecal lipids and reduced plasma triglycerides after intragastric fat challenge, while intravenous lipid infusion can cause liver steatosis in the knockout mice [143]. These results highlight the essential role of Parkin in regulating lipid absorption besides mediating mitophagy, which may contribute to the contrary effects of Parkin deficiency in alcohol and HFD-induced liver injury.

In addition, Bnip3-mediated mitophagy also plays an important role in regulating hepatic lipid metabolism and may be protective against NAFLD progression [136,144]. BNip3 knockout mice have increased lipid synthesis that is associated with decreased AMP-regulated kinase (AMPK) activity and increased expression of lipogenic genes. Moreover, levels of hepatic Bnip3 are markedly increased during fasting, which is associated with decreased β-oxidation of fatty acids in Bnip3 knockout mouse livers [144]. Sirtuin 3, a type of NAD-dependent deacetylase expressed mainly in mitochondria that promotes the expression of Bnip3 and Bnip3-mediated mitophagy via activating the extracellular-signal-regulated kinase-cAMP-response element-binding protein signaling pathway. Genetic overexpression of Sirtuin 3 protected against apoptosis in primary hepatocytes treated with PA [136]. Pharmacological activating Bnip3-mediated mitophagy alleviates steatosis in BRL (buffalo rat liver) cells, a cell line established from buffalo rat liver, treated with OA [141]. Peroxiredoxin 6 (PRDX6), an antioxidant PRDX family member that can translocate to damaged mitochondria, may also contribute to restoring hepatocytes’ mitophagy and maintaining mitochondrial function, exhibiting a protective role in NAFLD [135]. So far, the functional studies characterizing the mitophagy regulating pathway in NAFLD were mainly conducted in vitro. It would be interesting to uncover the in vivo hepatic mitophagy level at different stages of the disease and demonstrate the essential role of these signaling pathways in vivo.

It is notable that mitochondria undergo morphological and functional changes in response to metabolic inputs. In a 6-week methionine- and choline-deficient (MCD) diet-induced NASH mouse model, enlarged mitochondria and accumulated mitophagy intermediates were observed in the liver, suggesting the important role of mitochondrial dynamics and mitophagy in metabolic adaptation to nutrient influx [86]. Genetic depletion of fusion protein OPA1 in the liver restored mitochondrial morphological stasis and diminished the accumulation of mitophagy intermediates, eventually rescuing MCD diet-induced liver damage [86]. In addition to mitochondrial fission/fusion proteins, a circadian regulator, brain and muscle Arnt-like protein-1 (Bmal1), has been reported to regulate mitochondrial size and respiratory functions. Bmal1 loss-of-function can cause swollen mitochondria with diminished respiration and elevated oxidative stress, which is likely due to decreased expression of mitochondrial fission protein Drp1. Consistently, the restoration of hepatic Bmal1 activities in high-fat diet mice improved the metabolic outcomes of NAFLD [145]. However, there is still a debate over whether the mitochondrial architectural change is a cause or compensative response of NAFLD. The metabolic capacity of enlarged mitochondria and the role of mitochondrial dynamics in mitophagy should be further explored in NAFLD. Nevertheless, it seems that multiple pathways/players that regulate mitophagy in hepatocytes are working together to maintain hepatic mitochondrial mass and integrity, which have direct impacts on the development and progression of NAFLD/NASH.

### 4.3. Mitophagy in Drug-Induced Liver Injury

Mitochondria play a central role in regulating cell death and liver injury induced by various drugs [146,147]. The timely removal of damaged mitochondria is critical in protecting against drug-induced liver injury. Acetaminophen (APAP), a widely used antipyretic and analgesic drug in the United States, is safe at therapeutic doses, while an overdose can cause liver injury and acute liver failure in both humans and animals [148,149]. The hepatotoxicity of this drug is due to the generation of a reactive metabolite, N-acetyl-p-benzoquinone imine (NAPQI), which initially depletes liver glutathione (GSH) and subsequently forms adducts with cellular proteins [150]. In hepatocytes, mitochondrial proteins are the major binding sites for NAPQI to form protein adducts [151], which impair the ETC, causing electron leakage and elevated oxidative stress. The oxidant stress can induce mitochondrial peroxynitrite formation, which causes mitochondrial DNA damage and mitochondrial protein nitration, triggering mitochondrial permeability transition (MPT) and subsequent cell necrosis [152,153,154].

We recently demonstrated that APAP administration increases Parkin translocation to mitochondria with concurrently increased ubiquitination of mitochondrial proteins and mitophagy induction in mouse livers [155]. Given the central role of mitochondria in the pathophysiology of APAP-induced cell death, the removal of damaged mitochondria and mitochondrial protein adducts by mitophagy is feasible to be a defensive mechanism in the liver for promoting the recovery from APAP-induced injury. It has been demonstrated that pharmacological induction of autophagy by rapamycin almost completely eliminates APAP-induced liver injury in mice, whereas inhibition of autophagy by 3-methyladenine or chloroquine can further exacerbate APAP-induced hepatotoxicity [156,157]. Baulies et al. reported that mice with lysosome dysfunction exhibit a higher mortality after APAP overdose due to impaired fusion of mitochondria-containing autophagosomes with lysosomes [158]. These results imply the protective role of autophagy/mitophagy in attenuating the hepatotoxicity induced by an APAP overdose. Interestingly, we found that Parkin knockout mice are resistant to APAP-induced liver injury, while PINK1/Parkin double-knockout mice develop more severe liver injury with markedly decreased mitophagy after APAP administration compared with wild type mice [155,159]. This is likely because PINK1-mediated mitophagy still occurs in the absence of Parkin, which may compensate for the lack of Parkin. However, acute knockdown of Parkin accelerated APAP-induced liver injury in mice, inferring that under the acute knockdown of the Parkin time window the mice do not have sufficient time to adapt to the acute loss of Parkin [157]. Indeed, our recent work revealed that very low levels of mitophagy were detected in the PINK1/Parkin double-knockout mouse livers with or without APAP treatment, and the PINK1/Parkin double-knockout mice have the most severe liver injury compared with wild-type or either of the single knockout mice [159]. It should be noted that although PINK1/Parkin-mediated mitophagy serves as a protective mechanism against APAP-induced hepatotoxicity, other potential Parkin-independent pathways may still need to be further identified.

Mitochondrial fragmentation and a decreased expression of mitochondrial fusion proteins Mfn1, 2 and/or Opa1 were observed after APAP treatment in mouse livers and cultured rat hepatocytes [160,161]. Although the total level of fission protein Drp1 was increased in the liver following APAP overdose, less Drp1 was detected on the mitochondria fraction [161]. The role of Drp1 in regulating mitochondrial morphology in APAP-induced liver injury remains unclear. Since fragmented mitochondria are relatively easy to be removed by mitophagy, future work is needed to explore the role of mitochondrial morphological changes and mitochondrial dynamic proteins in APAP-induced mitophagy and liver injury.

### 4.4. Mitophagy in Liver Ischemia/Reperfusion Injury

Hepatic ischemia-reperfusion (IR) injury is one of the major complications of liver resection, transplantation, and hemorrhagic shock [162]. The loss of oxygen and nutrition depletion during ischemia leads to an ATP shortage in hepatocytes and non-parenchymal liver cells, which disrupts intra-cellular energy-dependent metabolic and transporting processes, resulting in accumulation of ROS and acidic metabolites [162]. The elevated oxidative stress and calcium overload promotes the high conductance permeability transition pores in the mitochondria to open and subsequently initiate the onset of MPT, leading to both apoptotic and necrotic cell death [163]. Although the acidic milieu suppresses numerous enzyme activities in the cytoplasm, during the acute ischemic period, the acidic environment confers protection to the liver parenchyma due to the inhibition of MPT [4]. Thus, the restoration of blood flow and pH returning to normal aggravates ischemic damage. Removing dysfunctional mitochondria through mitophagy has been reported as an important recovery mechanism in hepatic IR injury. Although nutrition deficiency is a powerful stimulus of autophagy, the nearly complete exhaustion of intracellular ATP during prolonged ischemia arrests this energy-dependent catabolic process. When reperfusion initiates, the reestablishment of the electron transfer chain and ATP synthesis in mitochondria enables autophagy to eliminate abnormal proteins and organelles that are produced during ischemia. Enhanced hepatic mitophagy is observed at the early stages of IR in both in vivo and in vitro models, which is associated with elevated cell death and aggregated liver injury [164,165,166]. However, the autophagy capacity also becomes impaired following prolonged ischemia, which may not be sufficient to eliminate the accumulated dysfunctional mitochondria. Therefore, at the late stage of reperfusion, the extended mitochondrial injury surpasses the ability of mitophagy, resulting in widespread MPT and cell death [162]. In C57BL/6 mice undergoing 60 min partial hepatic ischemia and 6 h reperfusion, mitochondrial biogenesis and PINK1/Parkin mediated mitophagy are diminished, while pharmacological stimulating mitophagy improves the IR outcomes, supporting the protective role of mitophagy in IR-induced liver injury [167,168,169].

Interestingly, severe liver injury is observed in aged mice following hepatic IR exposure compared to young mice, indicating that an aged liver responds differently to the stress [170]. Although the basal autophagy level is comparable in young and aged mice, the aged mice fail to remove damaged mitochondria after hepatic IR, showing defective SIRT1/Mfn2 mediated-mitophagy in the liver, which contributes to increased susceptibility to IR injury [171]. In human hepatic biopsy specimens, Parkin expression is negatively correlated with donor age and the peak level for aspartate aminotransferase is within the first week after liver transplantation, suggesting that PINK1/Parkin-mediated mitophagy is likely to be a protective mechanism in IR-induced injury [172]. In addition, alcoholic fatty liver may also be susceptible to IR injury as alcohol feeding accelerates hepatic IR injury in C57BL/6 mice, which is associated with restricted mitophagy [173]. The precise mechanism by which mitophagy protects against hepatic IR injury in an aged and alcoholic liver still needs to be further investigated.

### 4.5. Mitophagy in Viral Hepatitis

The hepatitis B virus (HBV) and hepatitis C virus (HCV) are hepatotropic viruses that infect millions of people globally, are responsible for a substantial portion of chronic liver diseases, and the death of approximately 1 million people around the world annually [174,175]. HBV and HCV share similar transmission modes that lead to similar pathological consequences including liver fibrosis, cirrhosis, and HCC. Accumulating evidence indicates that both HBV and HCV infection affects the general bulk autophagy and selective mitophagy.

HBV is an enveloped DNA virus with the main structural components of HBV including pre-S/S (HBsAg, the hepatitis B surface antigen), C (HBc/eAg, core/e antigen), P (polymerase, reverse transcriptase) and X (HBx). Overexpression of the entire HBV genome or HBx alone increases the formation of autophagosomes, which is dependent on the X protein, and directly binds with phosphatidylinositol 3-kinase to enhance its enzyme activity. It has generally been agreed upon that HBV utilizes autophagy to enhance its replication [176,177]. Interestingly, HBV infection induces mitochondrial fragmentation and mitophagy, likely serving as survival mechanisms by inhibiting cell death in HBV infected cells. Mechanistically, HBV infection increases the phosphorylation of Drp1 resulting in mitochondrial Drp1 translocation and mitochondrial fission. Moreover, HBV also increases the expression of PINK1 and Parkin as well as Parkin mitochondrial translocation to trigger mitophagy [178,179]. MARCH5 is an outer mitochondrial membrane E3 ligase, which is found to interact with HBx to promote the degradation of HBx protein aggregates via the ubiquitin proteasome system. As a result, overexpression of MARCH5 attenuates HBV-induced hepatic inflammation and mitophagy, and MARCH5 expression is positively correlated with the survival of HCC patients [180]. However, whether this declined mitophagy is due to less HBx protein or profound degradation of the mitophagy receptor FUNDC1 remains unknown. The beneficial effects of mitophagy against HBV-induced liver pathogenesis are further supported by the finding that thyroid hormone (TH) suppresses HCC development and protects hepatocytes from HBx-induced damage via increased PINK1-Parkin-mediated mitophagy [181].

HCV is a small enveloped RNA virus that possesses a single-stranded RNA genome, which encodes a single polyprotein that is processed into non-structural (ion channel p7, NS2, NS3, NS4A, NS4B, NS5A, and NS5B) and structural (core and envelope glycoproteins E1 and E2) proteins by host and viral proteases [182]. Several studies have shown that HCV infection can affect autophagy processes, although it seems controversial whether HCV can increase autophagic flux [183,184,185,186]. One early study reported that HCV infection increases the formation of autophagosomes by impairing the fusion of the autophagosome with the lysosome [183]. Later studies have suggested that HCV does increase autophagic flux but may be a time-dependent event as autophagy is less efficient at the early stage of HCV infection and becomes more efficient at later stages. [186,187,188]. Regardless of these controversial findings, it is generally agreed upon that HCV may use autophagosomes, perhaps as a membrane platform, to promote HCV replication.

In addition to the general autophagy, HCV infection can also affect mitophagy although it is controversial as to whether HCV increases or suppresses mitophagy [189,190,191]. Kim et al. reported that HCV infection not only increased the expression of Parkin and PINK1, but it also increased Parkin mitochondrial translocation and mitochondrial protein ubiquitination that led to mitophagy [189]. In contrast, Hara et al. reported that the HCV core protein directly interacted with Parkin and inhibits Parkin mitochondrial translocation after treatment with CCCP that resulted in decreased mitophagy [190]. The contradictory findings between these two studies could be due to the experimental conditions such as the presence or absence of CCCP in the culture and the different post-infection time. Subsequent studies from Kim et al. further found that HCV infection increased the phosphorylation of Drp1 and Drp1 mitochondrial translocation that led to mitochondrial fragmentation and mitophagy [191]. A more recent study showed that HCV non-structural protein 5A (NS5A) induced mitochondrial depolarization and fragmentation followed by mitophagy [192]. Currently, it is unclear whether NS5A can directly regulate the phosphorylation of Drp1 and promote its mitochondrial translocation or by affecting other kinases that may phosphorylate Drp1. Similar to HBV-induced mitophagy, it has been suggested that mitophagy may favor cell survival and promote HCV-mediated HCC development and progression. Indeed, ginsenoside Rg3 (G-Rg3) inhibits HCV-induced mitochondrial fragmentation and mitophagy by attenuating Drp1 phosphorylation and mitochondrial translocation, resulting in decreased HCV propagation [193]. Together these data indicate that targeting mitochondrial dynamics and mitophagy may lead to new therapeutic avenues for treating viral hepatitis.

### 4.6. Mitophagy in Liver Cancer

It has been generally accepted that autophagy acts as a tumor suppressor. Mice with a heterozygous deletion of Beclin 1 show decreased autophagy and increased cell proliferation, and later develop spontaneous tumors in multiple tissues, and the development of hepatocellular carcinoma induced by hepatitis B virus is accelerated [194]. Allelic loss of one essential autophagy-related gene Beclin1 has also been frequently observed in human breast, ovarian, and prostate cancers [195]. Because Beclin 1 has a BH3 domain, similar to Bcl-2 family proteins, it has been argued whether the lack of Beclin 1-induced tumorigenesis might be independent of its autophagy function. Subsequent findings from mice with liver-specific deletion of either Atg5 or Atg7 show spontaneous liver tumors, unequivocally indicating that autophagy is a bona fide tumor suppressor [196,197]. Multiple mechanisms have been identified that may contribute to the autophagy deficiency-induced liver tumorigenesis, including p62-mediated noncanonical Nrf2 activation, activation of Yap and mTOR, as well as the release the DAMP molecule HMGB1 [197,198,199,200,201]. Autophagy removes damaged proteins and organelles to protect cells from the intracellular oxidative and genotoxic stress, which prevents tumor initiation [202,203]. However, once the tumors have been formed, tumor cells also use autophagy to support their proliferation by providing substrates for mitochondrial metabolism to survive a general hash hypoxic tumor microenvironment [204,205]. Therefore, it is in general consensus that autophagy plays dual roles in both suppressing and promoting tumor development depending on the stage of tumorigenesis [204,206,207,208,209].

In the liver, hepatocytes are enriched with mitochondria and they comprise 13–20% of the liver volume [210]. Mitochondria are widely recognized as the main source of reactive oxygen species (ROS) production in mammalian cells. Physiological levels of ROS function as signaling molecules to modulate biological processes, whereas excessive ROS damage DNA, protein, and lipids, all of which have been etiologically implicated in the pathogenesis of human cancers [211,212,213]. The removal of dysfunctional mitochondria by mitophagy can relieve the intracellular oxidative stress, thereby protecting against hepatic tumorigenesis. As discussed above, in the HBV-encoded X protein (HBx)-induced mouse HCC model, the thyroid hormone was reported to decrease HCC incidence through activating the PINK1/Parkin pathway to induce mitophagy in hepatocytes, consequently reducing the ROS inflicted DNA damage [181]. Mitochondrial respiratory function and biogenesis were enhanced once mitophagy was activated in HepG2 cells treated with thyroid hormone triiodothyronine (T3). The expression level of PINK1 was restricted in human HCC tumor tissues compared to adjacent normal tissue, suggesting aberrant mitophagy activity in highly proliferative tumor tissues [181].

Furthermore, HCC is a well-known inflammation-related cancer as more than 90% of HCCs arise in the context of hepatic injury and inflammation [214]. When mitochondria become damaged, ROS and mitochondrial DNA (mtDNA) are released into the cytosol to activate the major innate immune response contributing to liver cancer initiation and progression [215]. Thus, mitophagy may add another barrier against HCC development by regulating the inflammatory response by clearing the accumulated dysfunctional mitochondria. Recently, Li et al. reported that FUNDC1-mediated mitophagy protects against liver carcinogenesis by inhibiting inflammasome activation [216]. In the diethylnitrosamine (DEN)-induced HCC mouse model, specific depletion of FUNDC1, a mitophagy receptor protein, in hepatocytes results in dysfunctional mitochondria accumulation, thereby triggering JAK/STAT signaling hyperactivation and inflammasome activation. Notably, the release of mtDNA further elevates a series of pro-inflammatory cytokines, resulting in a magnificent inflammatory response, and eventually promotes liver carcinogenesis [216].

Paradoxically, rapidly expanded liver cancer cells also require mitophagy to maintain their mitochondrial homeostasis, disruption of which may disrupt metabolism and elevate oxidative stress, leading to cancer cell apoptosis. Sesamol, a nutritional phenolic compound enriched in sesame seeds, can induce HepG2 cell apoptosis through inhibiting PI3K/Beclin-1 dependent mitophagy, which is linked to impaired mitochondrial function and accumulated H_2_O_2_ [217]. Similarly, inhibition of PINK1/Parkin-mediated mitophagy was regarded as an anti-tumor mechanism of several Chinese herb extracts (for example matrine and alantolactone) due to the promotion of apoptosis in HepG2 cells [218,219]. Moreover, PINK1-mediated mitophagy can inactivate mitochondrial-located tumor suppressor p53, to maintain the hepatic cancer stem cell (CSCs) population, which is thought to play important roles in HCC tumorigenesis [220]. When mitophagy is impaired, PINK1-activated p53 can translocate to the nucleus, which would otherwise be degraded by mitophagy, to suppress the expression of NANOG, a key transcription factor required for stem cell self-renewal, resulting in a reduced CSC population [220]. These findings suggest that mitophagy can sustain tumor cell metabolism and provide them with nutrients for tumor growth and survival, which is required for malignant tumor progression [221]. Overall, similar to general autophagy, it seems that mitophagy also plays a dual role in liver cancer development depending on the stage of tumorigenesis. Mitophagy prevents HCC initiation by suppressing dysfunctional mitochondria accumulation, cellular oxidative stress, genome instability and inflammation. Once carcinogenesis has been initiated, mitophagy is highly activated to support tumor cell metabolism demand and promote HCC progression. Therefore, a targeted therapy selectively suppressing mitophagy in actively proliferating tumor cells but enhancing mitophagy in adjacent normal cells may be a promising but challenging therapeutic direction for liver cancer in the future.

## 5. Analysis of Mitophagy in the Liver

So far, the most widely used methods to study mitophagy in mammalian cells include electron microscopy (EM), fluorescence microscopy for co-localization of mitochondria with autophagosomes or lysosomes, mitochondrial mass determination, and a series of newly developed pH-sensitive fluorescent probes [12]. EM is an excellent tool for observing mitochondria-containing autophagosome and autolysosome structures in various stages. However, due to the limited number of sections and cells, it is not easy to quantify mitophagy using EM. Also, a trained eye is required for proper identification of mitochondria, autophagosomes, and lysosomes under EM. Co-localization of mitochondria with autophagosomes and lysosomes through fluorescent co-labeling is able to provide results in a large number of cells, including live cells, for quantification. However, the fluorescence-labeled LC3 may aggregate and be misleading in some cases, and this assay requires superb image quality to be reliable, especially for tissue sections. Moreover, like all the other image-based approaches, only mitochondria-containing autophagic structures can be indicated in this assay but not the degraded mitochondria. The final degradation process of mitophagy can be determined by mitochondrial mass measurements, which include MitoTracker staining, mtDNA analysis, Western blot using antibodies against mitochondrial proteins, and citrate synthase activity assay. These assays are more objective and quantitative than previous image-based assays. However, MitoTracker staining relies on the normal mitochondrial membrane potential and cannot be conducted in fixed liver tissues. Mitochondrial proteins, particularly outer membrane proteins, are degraded by both autophagy and proteasome [34,41,222]. Therefore, when using Western blot or immunostaining to monitor mitophagy, multiple mitochondrial proteins, including the inner membrane and matrix proteins, should be applied.

The development of pH-dependent fluorescent molecular tools greatly improved the quality of traditional fluorescent imaging, making the image-based approaches more accurate and reproducible in monitoring mitophagy. More importantly, these assays can be used to monitor and quantify mitophagy in vivo in different tissues. Generally, in these molecular tools, both GFP and mCherry fluorescent proteins are tagged to a mitochondrial protein or a mitochondrial targeting sequence, forming a pH-sensitive probe being overexpressed in the mitochondria of target cells or tissues. The principle of this assay is based on the fact that the green fluorescence of GFP generally quenches, whereas mCherry fluorescence is relatively stable at the cellular acidic compartments (such as autolysosomes that have low pH values). Therefore, normal mitochondria generally show yellow color, whereas autolysosome-enwrapped mitochondria will only display red fluorescence. A transgenic mouse line that overexpresses Mito-QC, a tandem mCherry-GFP tag fused to the mitochondrial targeting sequence of an outer mitochondrial membrane protein, has been recently established to monitor mitophagy in various mouse tissues including the liver [223]. It should be noted that most outer mitochondrial membrane proteins are degraded by the ubiquitin proteasome system during mitophagy, whereas the degradation of inner mitochondrial proteins are more specific for mitophagy [12]. Nonetheless, Cox8-EGFP-mCherry, which targets the inner mitochondrial membrane, may be more specific than the Mito-QC that has been developed for monitoring mitophagy [159,224]. Although the pH-dependent fluorescent marker has great value for the monitoring and quantifying of mitophagy, it also has the limitations of many other imaging-based assays such as the lack of data robustness and consistency among different investigators scoring the mitophagy events. Another challenging issue that cannot be solved by the pH sensitive assays is to distinguish autophagosome-dependent (macroautophagy) and -independent mitophagy (microautophagy), the latter is achieved by lysosome only without autophagosome formation. Nonetheless, this property may provide an advantage as other methods will miss microautophagy and MDVs for mitochondrial turnover/quality control. The methods that have been reported to monitor mitophagy in liver diseases are summarized in Table 2.

## 6. Summary and Future Perspectives

**Figure 5:**
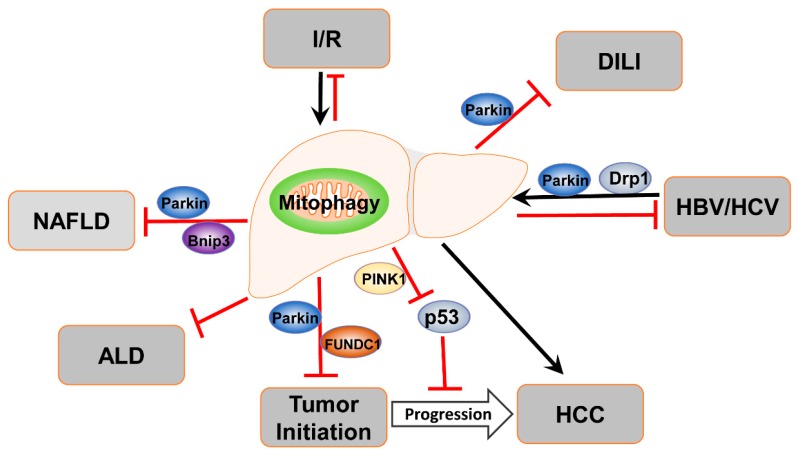
Summary of the role of mitophagy in liver disease. Mitophagy plays protective roles against drug-induced liver injury, the pathogenesis of ALD and NAFLD, as well as ischemia/reperfusion. In contrast, mitophagy may play dual roles in liver tumorigenesis and its progression. In viral hepatitis, mitophagy is highly activated, serving as a survival mechanism by inhibiting cell death in HBV/HCV infected cells.

In summary, current research progress has significantly advanced our understanding of molecular mechanisms involved in the regulation of mitophagy in liver pathophysiology. Mitophagy plays a protective role against drug-induced liver injury, the pathogenesis of ALD and NAFLD, as well as viral hepatitis. In contrast, mitophagy may play dual roles in liver tumorigenesis and its progression (Figure 5). Despite these progressions, many questions still remain. One future challenge for researchers is to validate these mitophagy pathways and identify quantitative autophagy and mitophagy markers in human liver samples of various disease contexts. While the PINK1-Parkin mitophagy pathway has been implicated in ALD, drug-induced liver injury, and liver cancer, the role of PINK1-Parkin-independent mitophagy pathway in liver pathogenesis has been under investigated. Moreover, the roles of the specific mitophagy receptors in the liver pathophysiology are also less known perhaps due to the lack of liver-specific knockout mice of these receptors. Several mouse lines have been introduced to study mitophagy in vivo including Mito-QC, the viral delivery of Cox8-GFP-mCherry to the mouse liver, and the newly identified mitophagy inducers from high-throughput screening. These have opened up more opportunities for future studies on mitophagy in liver diseases using these animal models. Ultimately these future studies may lead to the identification of new drugs that target mitophagy and provide better therapeutic options for treating acute and chronic liver diseases involving mitochondria dysfunction.

## Figures and Tables

**Table 1:** Identified mitophagy receptors in mammalian cells.

Receptor	Type	Interactor	Activated Conditions	Functions in Mitophagy	Refs
SQSTM1/p62	SAR	Ubiquitin	Mitochondrial depolarization	Recruited by Parkin-mediated ubiquitination, favoring mitochondrial cluster and recognition by the autophagy machinery and subsequent elimination	[42,43,44,68,91]
NDP52/OPTN	SAR	Ubiquitin	Mitochondrial depolarization	Recruited by PINK1 to mitochondria to activate mitophagy directly, independently of parkin	[68,92,93]
BNIP3	MAR	OMM	Hypoxia	Dual functions in regulating both cell death and mitophagy; Enhanced binding to LC3 when LIR motif is phosphorylated on Ser17 and Ser24	[51,52]
BNIP3L (NIX)	MAR	OMM	Hypoxia; erythrocyte maturation	Binds to LC3 mediating mitochondrial elimination during erythrocyte maturation; Ubiquitinated by Parkin to recruits other SARs (NBR1)	[45,53,54,55]
FUNDC1	MAR	OMM	Hypoxia	Recruits LC3 to initiate mitophagy; Binds to Drp1 to facilitate mitochondrial fission once activated	[57,84]
Bcl2-L-13	MAR	OMM	Mitochondrial depolarization	Stimulates mitochondria fragmentation and induces mitophagy through LC3 binding in HEK293 cells	[56,94]
FKBP8	MAR	OMM	Hypoxia	Recruits LC3A to mediate Parkin-independent mitophagy; Facilitates mitophagy by inducing mitochondrial fragmentation	[85,95]
NIPSNAP1/2	MAR	OMM	Mitochondrial depolarization	Mitochondrial matrix proteins, accumulating on the OMM following mitochondrial depolarization, recruiting autophagy receptors and adaptors	[96]
Ambra1	MAR	OMM	Mitochondrial toxins	Collaborates with E3 ligase HUWE1, binding to LC3 to induce mitochondrial clearance	[67,97]
PHB2	MAR	IMM	Mitochondrial depolarization	Activated upon proteasome-dependent OMM rupture	[46]
Cardiolipin	Lipid	OMM	Mitochondrial toxins	Externalizes to OMM and interacts with LC3 under mitochondrial stress in neuron cells	[61]
Ceramide	Lipid	OMM	Unknown	Binds LC3 to recruit autophagosomes to the mitochondria resulting in lethal mitophagy in cancer cells	[63]

Note: SARs, soluble autophagy receptors; MARs, membrane-anchored autophagy receptors; OMM, outer mitochondrial membrane; IMM, inner mitochondrial membrane.

**Table 2:** Methods monitoring mitophagy in liver disease.

Methods	Pros	Cons	Applications in Liver and Liver Disease Study
Electron Microscopy (EM)	Provides mitochondria-containing autophagosome and autolysosome ultrastructure	Limitations in quantification, steady-state rather than detecting flux	ALD [113,114,115,119] DILI [155,159] I/R [168] HBV/HCV [189]
Immunoelectron Microscopy (IEM)	Provides mitochondria-containing autophagosome and autolysosome ultrastructure and related proteins	Not quantitative	ALD [113,119] HBV/HCV [189]
Co-localization of LC3 with a Mitochondrial Protein	Large number of cells	The fluorescence-labeled LC3 aggregates may be misleading Not objective and robust Will not be able to detect LC3-independent mitophagy, microautophagy or MDVs	ALD [119,122] NAFLD [86,133,134,136,137,138,139] DILI [158] I/R [166] HBV/HCV [178,179,189] Cancer [217,218]
Autophagy/Mitophagy Marker Proteins	Objective Quantitative	Non-specific The intracellular distribution of marker proteins is more important than its total amount, total amount does not equal activity, only steady state	ALD [114,115,119,120,122] NAFLD [133,134,136,137,138,139,140] I/R [165,167,168,169,172,173] HBV/HCV [178,179,189,190,192] Cancer [216,217,218]
Mitochondrial Mass	Objective Quantitative	Only reflect steady state, rather than flux, nor the degradation or the initiation process of mitophagy. Mitochondrial outer membrane proteins are also degraded by proteasome.	ALD [114,116,119,122] NAFLD [134,140] DILI [155,157,159] HBV/HCV [192] Cancer [216]
pH-Sensitive Fluorescent Probe	Specific High image quality Apply in vivo and in vitro	The expression level of fluorescent proteins varies in different cells/tissues Not robust and easy to be dependent on individuals who are performing the quantification. Also, the half-life of the red puncta in the lysosomes may be dependent on cellular context and conditions, e.g., the activities of the lysosomal proteases	NAFLD [86] DILI [159] Cancer [216]

## List of Abbreviations

ALCAT1	Acyl-CoA:lysocardiolipin acyltransferase-1;
Ambra1	autophagy/beclin-1 regulator-1;
ALD	alcoholic liver disease;
AMPK	AMP-regulated kinase;
APAP	acetaminophen;
ATF5	Activating transcription factor 5;
Atg8	autophagy-related protein 8;
ATL3	atlastin GTPase 3;
Bcl2L13	Bcl2 like 13;
BNIP3	Bcl2/adenovirus E1B 19 kDa protein-interacting protein 3;
CCPG1	cell cycle progression 1;
CL	cardiolipin;
CSCs	cancer stem cells;
DAMPs	damage-associated molecular patterns;
DEN	diethylnitrosamine;
Drp1	dynamin-related protein;
ETC	electron transport chain;
FFA	free fatty acid;
FAM134B	family with sequence similarity 134, member B;
FUNDC1	FUN14 domain containing 1;
Gp78	glycoprotein 78;
G-Rg3	ginsenoside Rg3;
GSH	glutathione;
HBx	HBV-encoded X protein;
HBV	Hepatitis B virus;
HCV	hepatitis C virus;
HFD	high-fat diet;
HSP70	heat shock protein 70; I
R	ischemia-reperfusion injury;
LC3	microtubule-associated protein 1A/1B light chain3;
LIR	LC3 interacting region;
MCD	methionine- and choline-deficient;
MDVs	mitochondria-derived vesicles;
Mfn1	mitochondrial fusion protein 1;
Mfn2	mitochondrial fusion protein 2;
MPT	mitochondrial permeability transition;
Mst1	macrophage stimulating 1;
mtDNA	mitochondrial DNA;
NAFLD	non-alcoholic fatty liver disease;
NAPQI	N-acetyl-p-benzoquinone imine;
NASH	nonalcoholic steatohepatitis;
NBR1	BRCA1 gene 1;
NDP52	nuclear domain 10 protein 52 kDa;
NS5A	non-structural protein 5A;
Opa1	optic atrophy 1;
8-OHdG	8-hydroxydeoxyguanosine;
OA	oleic acid;
PA	palmitic acid;
PARL	presenilin associated, rhomboid-like;
PGAM5	mitochondrial phosphatase phosphoglycerate mutase family member 5;
PINK1	phosphatase and tensin homolog-induced putative kinase 1;
PHB2	prohibitin 2;
PRDX6	Peroxiredoxin 6;
ROS	reactive oxygen species;
RTN3	reticulon 3;
SARs	soluble autophagy receptors;
SQSTM1	Sequestosome 1 (SQSTM1);
TAX1BP1	TAX1 binding protein 1;
TEX264	testis expressed 264;
TH	thyroid hormone;
TOM20	translocase of outer mitochondrial membrane 20;
USP30	ubiquitin-specific peptidase 30;
USP15	ubiquitin-specific peptidase 15;
UPR^mt^	mitochondrial unfolded protein response;
VDAC	voltage-dependent anion channel.
